# The Connectome Visualization Utility: Software for Visualization of Human Brain Networks

**DOI:** 10.1371/journal.pone.0113838

**Published:** 2014-12-01

**Authors:** Roan A. LaPlante, Linda Douw, Wei Tang, Steven M. Stufflebeam

**Affiliations:** 1 Athinoula A. Martinos Center for Biomedical Imaging, Massachusetts General Hospital, Charlestown, Massachusetts, United States of America; 2 VU University Medical Center, Department of Anatomy & Clinical Neurosciences, Amsterdam, The Netherlands; 3 Department of Radiology, Harvard Medical School, Boston, Massachusetts, United States of America; 4 Harvard-MIT Health Sciences and Technology, Cambridge, Massachusetts, United States of America; Universiteit Gent, Belgium

## Abstract

In analysis of the human connectome, the connectivity of the human brain is collected from multiple imaging modalities and analyzed using graph theoretical techniques. The dimensionality of human connectivity data is high, and making sense of the complex networks in connectomics requires sophisticated visualization and analysis software. The current availability of software packages to analyze the human connectome is limited. The Connectome Visualization Utility (CVU) is a new software package designed for the visualization and network analysis of human brain networks. CVU complements existing software packages by offering expanded interactive analysis and advanced visualization features, including the automated visualization of networks in three different complementary styles and features the special visualization of scalar graph theoretical properties and modular structure. By decoupling the process of network creation from network visualization and analysis, we ensure that CVU can visualize networks from any imaging modality. CVU offers a graphical user interface, interactive scripting, and represents data uses transparent neuroimaging and matrix-based file types rather than opaque application-specific file formats.

## Introduction

A foremost aspiration in neuroscience is understanding the connectivity of the human brain. Recent years have seen considerable growth of interest in the human connectome, the effort to map the entire connectivity of the human brain. The analysis of this high-dimensional connectivity data presents a particularly challenging problem. The emerging field of connectomics has risen to meet this challenge, leveraging new advances in graph theory to understand human brain function and leading to the development of multi-institution initiatives such as the Human Connectome project [1].

The field of connectomics began with work on the *C. Elegans*, a roundworm whose neural connectivity was completely characterized, creating a network with 302 neurons [2]. In a seminal discovery about the complex organization of graphs, the nervous system of *C. Elegans* as well as other complex networks from numerous domains were shown to exhibit small-world organization [3]. Graph theoretical approaches lending new insight to the dynamics of complex networks began to proliferate, including in the study of social networks, the internet, and food webs [4].

More recently, the study of complex brain networks by analysis of graph theoretical properties has become exceptionally popular. In addition to small-world organization, whole brain networks have shown various nontrivial properties, including scale-free distribution [5], rich-club organization [6] and the hierarchical organization of networks on multiple scales [7], [8].

Graph theory has led to insights on clinical populations. In epilepsy, small world organization is reduced during ictal activity compared to interictal activity [9], and also reduced at rest compared to healthy controls [10], The default mode network in epilepsy patients also shows altered structural connectivity compared to healthy controls [11], [12]. Whole brain networks have also demonstrated applications to schizophrenia [13], [14], Alzheimer's disease [15], attention-deficit/hyperactive disorder [16], and personality traits [17].

Mapping the human connectome non-invasively is currently being pursued using multiple imaging technologies. Connectomics has focused most closely on resting state fMRI [18] and diffusion MRI [19]. However, network studies of resting state electrophysiological recordings such as electroencephalography (EEG) and magnetoencephalography (MEG), which directly measure neural currents at high temporal resolution [20]–[22], as well as networks derived from cortical thickness measurements [23], have also been used. Many different analysis techniques exist to estimate connectivity in the brain in each of these modalities.

In order to map the human connectome, sophisticated visualization and analysis tools are required. A wide variety of free and open source software packages for the analysis of neuroimaging connectivity data are readily available. Most software packages currently available are integrated brain network tools that are designed for processing a single imaging modality Such packages include Camino [24], MNE [25], [26], dipy [27], Brainstorm [28], the Connectome Mapper [29], the MR Connectome Analysis Pipeline [30] and many other unpublished packages. As these packages are typically specialized for processing a particular modality, they have only limited visualization features, and are not necessarily designed to analyze multi-modal data.

There are a number of software packages designed for the visualization and analysis of domain-general complex networks, such as Pajek [31] and Gephi [32]. Notably, there are several software packages specially designed for visualization and analysis of the human connectome: the Connectome Viewer [33], the BrainNet Viewer [34], and the VisualConnectome toolbox (http://code.google.com/p/visualconnectome). Building on visualization features from these packages, we sought to provide new interactive features for the visualization of structural and functional connectivity, and make visualization a more interactive process.

Here, we introduce a general-purpose, free, and open source software package written in python specially designed for the visualization of multi-modal human brain networks: the Connectome Visualization Utility (CVU). CVU is a standalone application with an intuitive graphical user interface (GUI) that automatically generates powerful, interactive visualizations of human brain networks from common matrix and imaging file formats. It is decoupled from the process of connectivity estimation and network creation, and can therefore be easily used to visualize networks from any imaging modality. CVU is designed with the visualization of complex graph theoretical properties in mind and allows network statistics to be easily incorporated into the visualization.

In this manuscript, we describe the features, usage, implementation and software dependencies of CVU. We illustrate the power of CVU's visualizations with example networks constructed from data from the Human Connectome Project. In the following section, we will briefly review the practice and findings of network analysis of human brain networks. As CVU is a visualization tool that requires a fully formed network as input, we will describe some schemes for creating brain networks and provide a limited review of other software packages that perform the network construction and can be used alongside CVU. Finally, we will review the advantages and limitations of CVU, and describe possible future directions for research.

## Methods and Results

**Figure 1:**
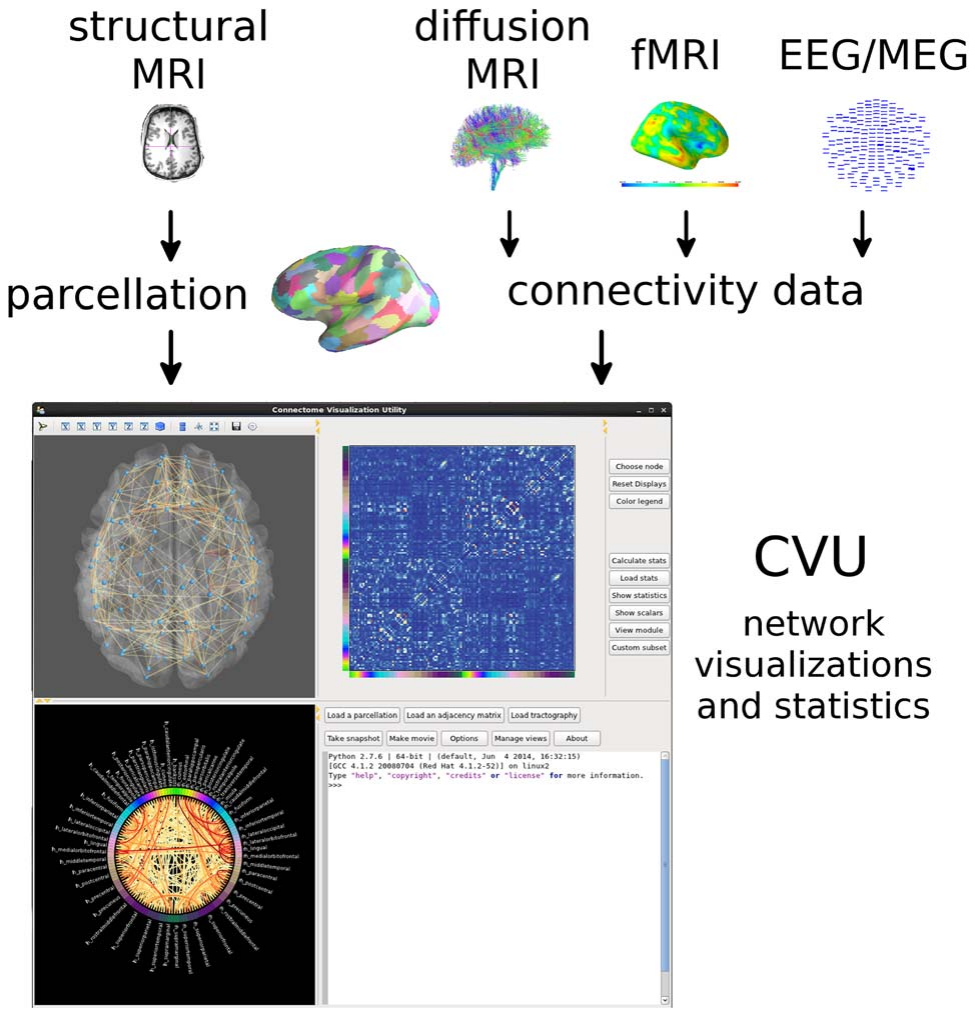
Basic workflow to create visualizations in CVU. The cortical surface is parcellated into ROIs, while connectivity matrices can be constructed from a variety of imaging modalities. The parcellation and matrix are both loaded into CVU via the graphical user interface, shown at the bottom.

CVU is an integrated application designed for the interactive visualization of human brain networks. Here we illustrate the features of CVU with sample fMRI, diffusion MRI, and MEG datasets, show the software dependencies, and suggest how to integrate CVU into an analysis workflow. The basic workflow is shown in Figure 1.

### 1. Implementation

Human brain networks in CVU are represented by two main components: an *adjacency matrix* and its accompanying *parcellation*.

In CVU, complex networks are represented internally as adjacency matrices. These matrices can be specified as any one of a variety of common matrix formats, including MATLAB (The Mathworks, Natick MA) and numpy [35] formats, as well as plain text files. Most work on human brain networks has focused on undirected (symmetric) connectivity – for simplicity, in current versions of CVU, all networks are represented as undirected.

Cortical and subcortical parcellations provide the ROIs that are used as network nodes in CVU. In CVU, parcellations are displayed as nodes located on or near the cortical surface. It is possible to specify parcellations in three ways: as parcels of surface vertices, collections of voxels, or ROI coordinate centers described in a stereotaxic space. Parcellations are specified by specifying a *surface* file and several *parcellation* files depending on the method. Surface files store geometric information about the morphometry of the cortex, can be created using FreeSurfer, an open source imaging analysis suite (http://surfer.nmr.mgh.harvard.edu/), and can be specified in either the native FreeSurfer format or the more general GIFTI format. In order to display the parcellation on the surface, it is necessary (but not required to run the program) to additionally provide a 4×4 registration matrix in any matrix format between the parcellation volume and the anatomical surface space.

Because annotation files representing surface parcellations typically only cover the cortical surface, in order to specify the locations of subcortical structures, a segmentation file can additionally be specified. The segmentation file is an MRI volume with specific hard-coded values to indicate the locations of numerous cortical and subcortical strutures. Segmentation is done using FreeSurfer [36] and the segmentation file can be provided in native FreeSurfer or other imaging formats such as NIFTI.

The identity of the ROIs in the matrices is determined by plaintext *ordering files* which accompany data in CVU. Ordering files are text files with one ROI name on each line. The first row and column of the matrix correspond to the region listed on line 1 of the corresponding ordering file, and so on. The location of the ROI in anatomical space is then determined by identifying the same node as specified in the parcellation. If the ROI name corresponds to a predefined set of subcortical structures, its location is determined by examining the segmentation file instead.

As CVU is a tool for visualization of abstract networks, visualization of individual subject morphology is not always a priority. CVU is designed so that subject specific morphology can be used if necessary; however, for most use cases, an average brain is preferred due to its lower speed and memory requirements, and simpler visualization. For convenience, CVU provides surface and segmentation files for the fsaverage5 brain, as well as a number of parcellation schemes. Specifically, annotations are provided for the multi-resolution surface parcellations described in [37], as well as the Destrieux and Desikan reconstructions automatically generated by FreeSurfer reconstruction [38], [39].

For parcellations of more than a few nodes, visualization of the entire network with many connections does not produce an easily interpretable result. Furthermore, at very high network resolution visualization can become quite slow, though this depends on the hardware. For these reasons, CVU automatically cuts off all but the strongest 500 connections in order to balance visualization and performance, while still offering control of these parameters to the user so that more connections can be shown if desired.

While CVU contains a graphical user interface through which users can interact with and load data, it also contains a scripting window and a scripting mode so that advanced users can leverage the power of python and interact with the underlying data structures and visualizations directly.

### 2. Visualization

Determining the best visualization for a complex network may be difficult due to the high dimensionality of the data. In graph theory, the two most common types of visualizations are *node link diagrams*, which display links between nodes as lines of arbitrary orientations, and *matrices*, in which nodes comprise the rows and columns while the matrix entries correspond to links. The best visualization depends on the structure of the dataset and the desired task – it has been argued that node link diagrams are more intuitive for graphs with few nodes, but are inferior to matrix representations when the number of nodes is large [40].

To reduce the complexity of visualizing high-dimensionality data, CVU provides three types of interactive, integrated visualizations: the *3D Brain* view, the *Matrix* view, and the *Circle* view. These three visualizations are designed to offer complementary overviews of the same data, so as to maximize the amount of information that can be accrued at a glance. The views are linked, and update simultaneously when the data changes. The complexity of the data is further reduced by a number of interactive features, which can further expand the number of possible views by isolating certain connections or by rotating the 3D brain. The three main views are presented directly to the user when the program is launched or when new connectivity data is supplied, with no intermediate scripting necessary. If the user launches the program without specifying any data, a sample dataset showing MEG synchronization likelihood connectivity [41] from one subject is loaded automatically. The GUI, as it appears immediately upon launching the program with no data, is shown in Figure 1.

**Figure 2:**
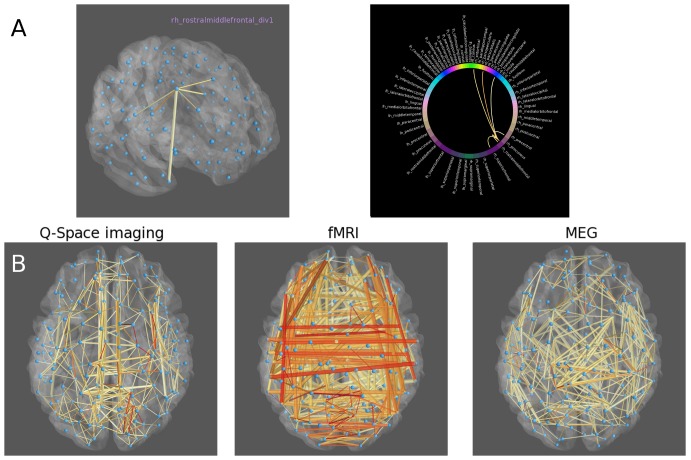
Visualizing networks in CVU. A) Clicking on a node in the network will show only isolated connections to that node. Thresholded connectivity to a rostral middle frontal ROI is displayed. B) Networks from a single subject and constructed from three different imaging modalities are shown. Topological differences in the three networks are immediately apparent upon visualization. Stronger connections are shown in red while weaker connections are shown in yellow.

In the 3D Brain view, a transparent cortical surface is displayed along with a node link representation, in which nodes are located in a realistic spatial scale inside the brain. This view is similar to visualizations found in the Connectome Viewer toolbox [33] and the UCLA Multimodal Connectivity Database [42], and has novel interactive features including the ability to interactively isolate connections – for instance, left clicking on a node will show only the connections to that node (see Figure 2a) and the node's label will be displayed.

In some cases the visualization of networks alone can lend insight into complex topological properties. Example multi-modal networks for a single subject from the Human Connectome Project are shown in Figure 2b. Networks were constructed from high b-value generalized Q-space diffusion imaging [43] using fiber counts, fMRI using correlation, and MEG using synchronization likelihood [41]. The fMRI network is characterized by the predominance of lateral connections between homologous regions. By contrast, the diffusion and MEG networks have less lateral connectivity, instead consisting mostly of short range connections. Depending on the analysis constraints, with visualization it is sometimes possible to determine some properties of the data, such as the modality of acquisition, solely by inspection. Accordingly, visualization of the network can serve as an important quality control before performing further statistical analysis.

**Figure 3:**
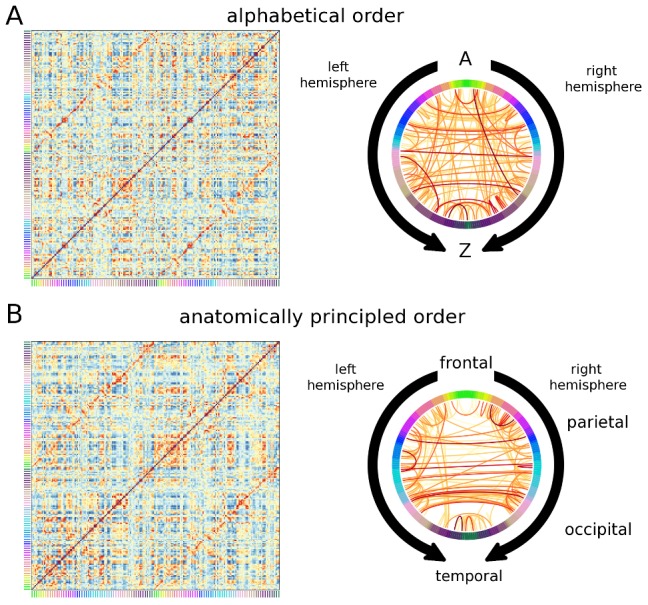
Effect of ROI order on visualization. A) Matrix and circle views for a single subject matrix constructed from correlations between BOLD signals, using an unprincipled ordering with ROIs ordered alphabetically by region name. Stronger connections are shown in orange while weaker connections are shown in blue. The node's anatomical grouping is given by the colors on the sides on the matrix. B) The same network shown using a principled anatomical ordering, beginning in medial frontal cortex, wrapping around parietal and occipital cortex, and ending at temporal pole. When the principled ordering is used, gains are especially seen in the circle view as short-range connections are grouped together to produce less visual clutter. Stronger connections are shown in dark red while weaker connections are shown in yellow. The colors on the circumference represent the same node identities as in the matrices.

In the matrix and circle views, nodes are positioned along the sides of a square matrix, or on the circumference of a circle, respectively (see Figure 3). Matrix entries and arcs between circle nodes are used to represent connections. Circular ideograms similar to the circle view exist in the open source software packages MNE python [25] and Circos [44]. Both the matrix and circle views are affected by the order in which the nodes are positioned. Figure 3 shows different visualizations of the fMRI network from figure 2 with two different node orderings. The first ordering is an unprincipled organization in which nodes are ordered alphabetically by region name; the second shows nodes ordered by anatomy, beginning in the frontal lobe, wrapping around the parietal and occipital lobes, and ending at the temporal pole. The anatomically principled ordering shows the structure of the dataset – primarily comprised of connections with strong lateral connectivity – much more clearly.

Each of the three views – the 3D brain, connectivity matrix, and connectivity circle, is updated simultaneously whenever parameters of the visualization change. Any of the three views can be exported to high resolution images for figure generation. In addition, the 3D brain can also record movies which capture user-defined interactive camera movements and can also optionally include a simple animation to rotate the displayed brain.

### 3. Network statistics

Complex networks are described by quantitative graph theoretical properties. To characterize these networks, various network statistics are computed, such as the clustering coefficient [3], local efficiency [45], centrality [46], [47], and modularity [4], [48]. CVU natively calculates and displays a range of weighted and binary, nodal and global network statistics. These measures can then be displayed topographically on the network, affording an overview of statistical patterns throughout the whole brain at a glance. Statistics computed by CVU can also be saved to disk as common matrix file formats. Any scalar metrics which CVU does not calculate natively can additionally be imported and displayed inside the program.

**Figure 4:**
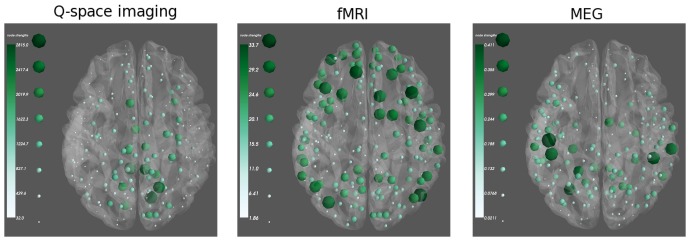
Visualizing scalar data in CVU. Node strength (weighted degree) is shown for the thresholded networks shown in Figure 2. The node strength corresponds to the size and color of the node. Nodes marked by large dark green spheres have high strength, and small light green spheres represent lower strength.

An example of network statistics calculated for multi-modal networks is shown in Figure 4, using the same networks from Figure 2. Networks for a single subject were constructed from resting state fMRI, high b-value Q-space diffusion imaging, and resting state MEG. The weighted degree of each node is displayed as the size and color of the sphere in the node, although it is also possible to use the size and color to represent two unrelated variables. Networks constructed from these different modalities show highly different topology: the most important nodes in the diffusion network are clustered around the white matter tracts in cingulate cortex, while in the resting state fMRI and MEG networks the strongest nodes are distributed widely throughout different areas of the neocortex.

One statistic of particular interest in visualization is the modular structure of brain networks. In order to calculate modular structure, CVU utilizes an algorithm to automatically identify the optimal modular structure by iteratively maximizing modularity [49], which is effective even when the optimal number and size of communities is not known. A fine-tuning algorithm to further optimize the modularity is then applied [50].

**Figure 5:**
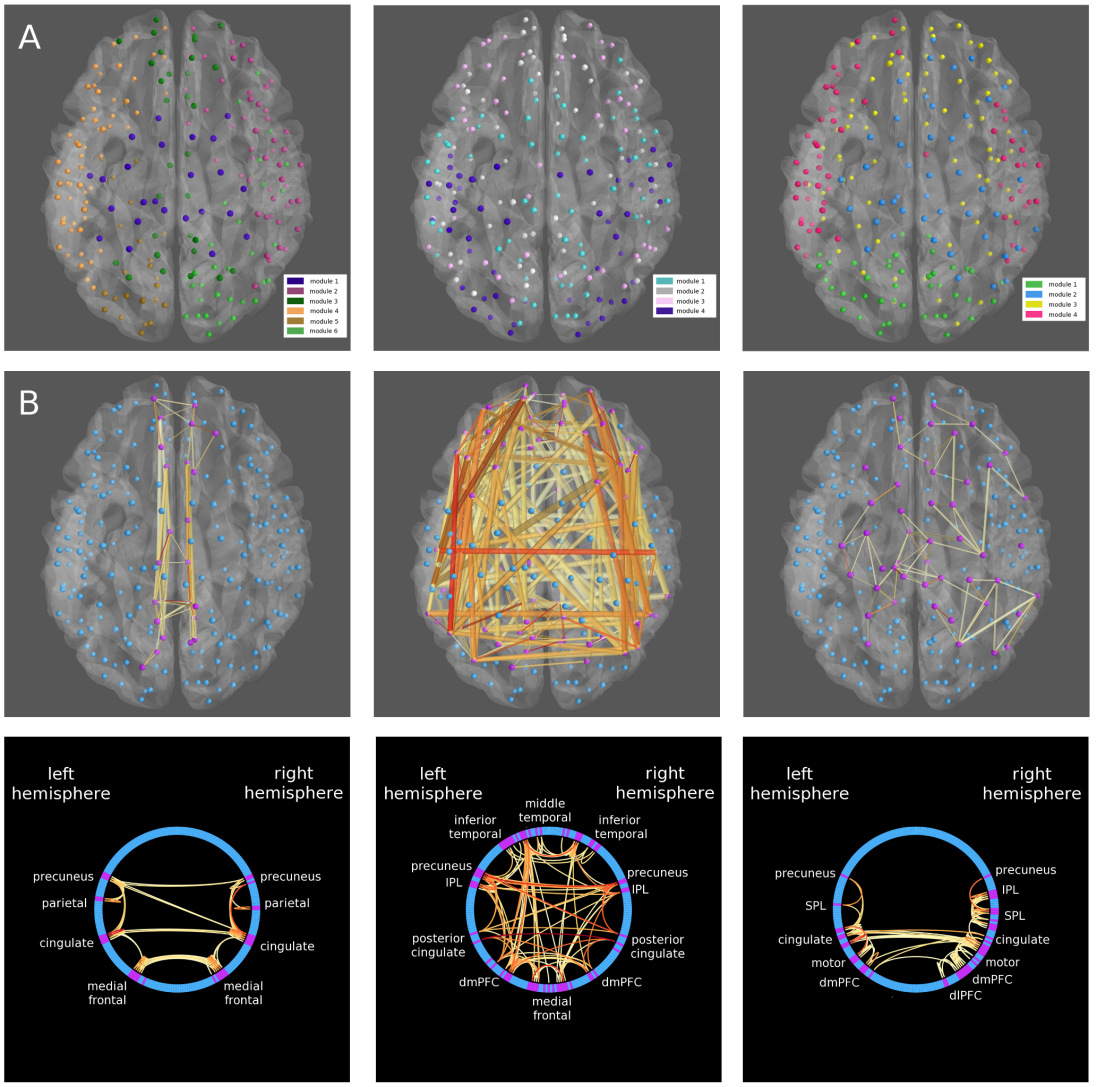
Visualizing modular structure in CVU. A) For the same three single-subject networks shown in Figures 2 and 4, modular structure is determined using a spectral modularity maximization algorithm. The resulting modules are displayed in different randomly selected colors. The order of modules is randomized. B) The module corresponding most closely to the default mode network is isolated in each network and shown in both the 3D brain and circle views. Only connections with both ends in the module are displayed. Stronger connections are shown in red while weaker connections are shown in yellow. Nodes inside the default mode module are shown in purple while other nodes are shown in blue.

Modular structures for the multi-modal networks shown in Figures 2 and 4 is displayed in Figure 5. In figure 5A, all modules found by a modularity maximization algorithm are displayed. In 5B, nodes in each modality from a single, automatically generated module corresponding closely to the default mode network [51] are isolated. Notably, the modules from Q-space diffusion imaging and MEG do not show perfect overlap with the classic default mode network, and especially so in the MEG network where the module contains sensory and motor regions. However, this result is not unexpected. Although previous attempts to isolate the default mode network using resting state MEG have been variously successful, they show that in MEG the default mode network is transient and that cross-hemispheric connectivity in homologous regions is sparse at frequencies above 1–2 Hz [52], [53].

To calculate network measures, CVU uses bctpy, the Brain Connectivity Toolbox for python. Bctpy is our group's translation of the Brain Connectivity Toolbox [54], a rich open-source library written in MATLAB for the analysis of brain networks. In contrast to the Brain Connectivity Toolbox, bctpy is written in pure python, alleviating the need for a nonfree MATLAB license and allowing the easy integration of network analysis into a python workflow without introducing additional dependencies.

### 4. Technical details

CVU depends extensively on other tools written in python. For interactive 3D visualization, CVU depends on the Mayavi library [55]. For 2D interactive plotting, CVU uses the chaco (http://code.enthought.com/projects/chaco) and matplotlib [56] libraries. The GUI is written using the traitsui framework (http://code.enthought.com/projects/traits_ui/) and supports both the Qt and wxwidgets toolkits.

Installation of CVU requires the installation of a scientific python distribution such as Enthought Canopy (http://www.enthought.com) or Anaconda (https://store.continuum.io/cshop/anaconda/) which satisfies most of CVU's dependencies. CVU has been tested with both Anaconda and Enthought Canopy and works fully in both python environments. After installing the scientific python distribution, it is necessary to install CVU as well as three additional dependencies: bctpy, MNE-python [26], and nibabel (http://www.nipy.org). Use of an installer such as ‘easy-install’ from python's setuptools modules is recommended to install CVU and its dependencies. A launcher script is provided alongside the source code to run the program.

CVU additionally has two optional dependencies. First, the cross-platform audio/video transcoding program FFmpeg (http://www.ffmpeg.org) is needed for creating movies from the 3D brain. Second, FreeSurfer is needed to define surface parcellations and create subject-specific surfaces, but is not needed to run CVU.

CVU is cross-platform software. CVU has been tested and runs on Linux, Mac OS X, and Windows. Almost all features in CVU are fully functional across platforms; however, creating movies from the 3D brain is only possible on Linux and Mac OS X systems running the X window system.

CVU can be scripted interactively, either by entering valid python code into the interactive shell, or by providing standalone scripts written in valid python. Standalone scripts are run by providing the script as an argument to CVU's launcher script. In addition, standalone scripts can be called interactively from the shell. These scripting features operate on the internal data structures directly, with several scripting helper functions additionally available for common operations, (e.g. loading a new adjacency matrix). Instructions on how to perform many useful operations in scripting mode are included in the documentation.

### 5. Software License

CVU is licensed under the GNU General Public License version 3, or optionally any later version. This is a free software license, such that CVU may be freely redistributed and modified by any party, and may also be used in commercial applications. However, when distributing the software, the imposition of any restrictions on any further redistribution is prohibited.

### 6. Ethics statement

The sample data shown in this manuscript was collected with the approval of the Massachusetts General Hospital IRB. Subjects gave written informed consent, and the Massachusetts General Hospital IRB approved the consent procedure.

## Discussion

In this paper we have described the Connectome Visualization Utility, a free and open-source software package dedicated to the visualization of the human connectome. CVU offers three different complementary interactive visualizations that efficiently depict complex brain networks of any scale and modality, as well as an easy-to-use GUI, a host of well-documented visual customizations for interactive data exploration using graph statistics and modular structure, and interactive scripting that allows direct access to the data and can leverage the tools available in a scientific python environment. CVU uses transparent data formats, representing data as the user's choice of common file types such as MATLAB matrices, text files with matrix data, and FreeSurfer or GIFTI surfaces.

### 1. Strategies for data collection

CVU is a software package for the visualization and network analysis of human brain connectivity data, but does not itself perform the connectivity estimation. To use CVU, it is necessary to first have some connectivity data. In this section, we describe some strategies for creating connectomes, and describe some other software packages which perform the connectivity estimation which can subsequently be used by CVU.

#### 1.1 Strategies for cortical and subcortical parcellation

Optimal parcellation of the brain for the construction of connectivity estimates is a difficult problem. However, several innovative solutions exist. The Destrieux and Desikan reconstructions [38], [39] can be generated automatically using FreeSurfer and divide the cortical surface into parcels based on macroscopic cytoarchitectonic and gyral profiles. These parcellations have been further subdivided into finer regions in order to generate 998 regions of roughly equal size [37]. As the spatial scale of parcellation is thought to affect the topology of the network, [57]–[60], these 998 regions were subsequently reassembled into several parcellations of varying scale, containing 998, 483, 241, 133, and 66 nodes respectively [61], which are available from the open source Connectome mapper [29]. These parcellations, registered to the FreeSurfer average brain “fsaverage5,” are included with CVU for convenience.

Other parcellation schemes are also possible. Some alternatives to anatomical parcellation include the clustering of functional activations [62]–[64] and clustering of white-matter bundles from diffusion tractography [65], [66]. While these schemes may overcome the ad hoc nature of anatomically parcellating functional data, the limitations of the choice of parcellation are not fully known [67], [68].

CVU is decoupled from any choice of parcellation, and thus is easily adaptable to a variety of parcellation schemes. CVU is able to represent parcellations defined as a collection of surface labels or as structures defined in a volume. It is also possible to represent the parcellation simply as a matrix file specifying 3-dimensional coordinates (e.g., EEG electrodes), optionally providing an additional registration matrix to orient these coordinates to the surface. Parcellations can be specified as any of multiple common file formats – surface-based parcellations may be represented with FreeSurfer or GIFTI annotation files, while volumetric parcellations can be specified with virtually any image format.

#### 1.2 Estimation of Structural Connectivity

Much work on the human connectome has focused on structural connectivity, using diffusion MRI to characterize the white matter connections [19], [69]–[71].

Matrix creation from white matter reconstruction typically employs a fiber counting strategy, where connectivity values consist of counts of tracts between finely parcellated regions of interest, normalized for differences in ROI size and distance [37]. Another technique is to estimate connectivity as the average or integral of a metric over the course of white matter tracts – for instance, the average magnetization transfer values over the streamline tracts connecting ROIs [72].

There are numerous open source software packages available for the processing and construction of diffusion data and the construction of diffusion brain networks using fiber counting algorithms, including Camino [24], the Connectome Mapper [29], DSI Studio (http://dsi-studio.labsolver.org/) and the dipy project [27].

#### 1.3 Estimation of Functional Connectivity

The functional connectome is characterized by statistical regularities between spontaneous neural activity in different regions, typically collecting in the resting state. Resting state fMRI is a common choice to examine functional connectivity because resting state fMRI networks are robust across subjects, such as the default mode network which disappears during task activity, as well as other networks including the ventral and dorsal attentional networks, the somatosensory networks, and others [51], [73], [74]. Resting functional connectivity can easily be calculated by correlating ROI timeseries produced by open-source software packages such as AFNI [75], FSL [76], and the FS-FAST toolbox in FreeSurfer.

Resting state from direct bioelectric signals, EEG and MEG, has gained a renewed interest, in part due to the analysis techniques from BOLD fMRI. The high temporal sensitivity of electrophysiological data allows the direct exploration of connectivity as cortical oscillations [77]. Common metrics used to capture spectral regularities between signals include phase-locking value [78], imaginary coherence [79], phase-lag index [80], [81], as well as many others [82]. In order to more closely approximate resting networks found in fMRI, novel techniques for network construction from EEG and MEG data using correlations and statistical analyses of power envelopes have additionally been developed [83]–[86]. MNE python [25], Brainstorm [28], and Brainwave (http://home.kpn.nl/stam7883/brainwave.html) are open-source software packages with the ability to calculate many spectral connectivity metrics from EEG and MEG data.

#### 1.4 Overview

CVU has been tested with networks generated with all of the software packages described above. By separating the process of network construction from the visualization, we ensure that CVU is able to work with networks constructed from any modality of data, generated from any software package, and using any algorithm.

### 2. Features, future directions and limitations

Interactive and flexible visualization is an important part of scientific discovery. Visualization can stimulate new hypotheses, allow researchers to quickly assess their findings at a glance, and serve as important quality control. As a network visualization tool, CVU performs all of these functions. In addition, CVU has novel interactive visualization features not available in existing software packages for visualization of the connectome such as the Connectome Viewer [33], such as the ability to interactively isolate connections to explore the network topology. CVU offers the ability to easily visualize network statistics. CVU can visualize modular partitions of brain networks, allowing comparisons to well-studied brain networks (e.g., default mode network, ventral attention network, etc.).

Further directions for research and software development may involve the use of new visualization strategies or improvements to existing visualizations. Incorporating useful visualization features from other software packages, such as interactive annotations, into one single software package is a possible direction of future software development. The development of novel visualization methods for interpreting complex networks is an important direction of future work. Development of new software that makes use of existing algorithms in scientific visualization is also important. For instance, some representations of circular ideograms similar to the circle view in CVU utilize a hierarchical bundling strategy, in which similar edge splines are clustered together, reducing visual clutter [87]. However, we are only aware of one software implementation of this technique – in the Functional Brain Connectivity Explorer [88], a tool with many similarities to CVU. The Functional Brain Connectivity Explorer, however, is only intended for the analysis of resting state fMRI data and requires volume-based brain atlases.

CVU is limited in the character of the networks that can be visualized. Only single-edged, undirected networks can be visualized. Methods of effective connectivity, corresponding to visualizations of directed graphs, are not currently supported. Likewise, multigraph networks which have multiple values per edge (e.g., EEG/MEG networks with separate edges for each frequency band) are not supported. Moreover, CVU's networks are static; no visualizations exist to depict the robustness or dynamic natures of networks collected from modalities such as EEG/MEG. The development of new easily interpretable visualizations for directed networks, multigraphs, and dynamically changing network structures is an important direction of future work.

Another limitation of CVU and a potential direction of future work is the inability to represent networks from multiple modalities or include network attributes in a single file, as in the Connectome Toolkit where data is stored in the XML-based Connectome File Format [33]. Other file formats such as the recent CIFTI-2 specification utilize similar flexibility. However, the choice to use regular matrix file formats instead possesses several benefits: use of more general file formats reduces software dependencies and ensures data transparency. Data transparency facilitates understanding of the software design and ensures that it is easy for users to create connectivity data using any methods, and additionally simplifies the identification of software bugs. CVU can natively and quickly calculate many common network metrics that might be stored in larger connectome files, and any attributes that CVU cannot generate natively could be saved individually in multiple files. The inclusion of whole-connectome inputs in future versions of CVU is a potential direction of future development, as more analysis tools are developed to make use of formats such as CIFTI-2.

### 3. Conclusion

In summary, CVU is a powerful free and open source visualization tool for the exploration and analysis of the human connectome that expands upon visualization features available in other software packages. It is able to leverage powerful tools available for neuroscientific analysis in a scientific python environment, to which it also represents an important contribution.

CVU can be downloaded at https://pypi.python.org/pypi/cvu. Extensive documentation is available at https://github.com/aestrivex/cvu/wiki.
